# Vision-Based Methods for Food and Fluid Intake Monitoring: A Literature Review

**DOI:** 10.3390/s23136137

**Published:** 2023-07-04

**Authors:** Xin Chen, Ernest N. Kamavuako

**Affiliations:** 1 Department of Engineering, King’s College London, London WC2R 2LS, UK; xin.7.chen@kcl.ac.uk; 2 Faculté de Médecine, Université de Kindu, Site de Lwama II, Kindu, Maniema, Democratic Republic of the Congo

**Keywords:** intake monitoring, drinking action detection, dietary monitoring, vision-based methods

## Abstract

Food and fluid intake monitoring are essential for reducing the risk of dehydration, malnutrition, and obesity. The existing research has been preponderantly focused on dietary monitoring, while fluid intake monitoring, on the other hand, is often neglected. Food and fluid intake monitoring can be based on wearable sensors, environmental sensors, smart containers, and the collaborative use of multiple sensors. Vision-based intake monitoring methods have been widely exploited with the development of visual devices and computer vision algorithms. Vision-based methods provide non-intrusive solutions for monitoring. They have shown promising performance in food/beverage recognition and segmentation, human intake action detection and classification, and food volume/fluid amount estimation. However, occlusion, privacy, computational efficiency, and practicality pose significant challenges. This paper reviews the existing work (253 articles) on vision-based intake (food and fluid) monitoring methods to assess the size and scope of the available literature and identify the current challenges and research gaps. This paper uses tables and graphs to depict the patterns of device selection, viewing angle, tasks, algorithms, experimental settings, and performance of the existing monitoring systems.

## 1. Introduction

Maintaining healthy food intake and adequate hydration is significant for humans’ physiological and physical health [1,2,3].

The quality of food intake was proven to be associated with the metabolic function of the human body [4]. Unbalanced nutrition intake increases the risk of many diseases, including diabetes, obesity, cardiovascular disease, and certain cancers [1,5]. When understanding human body dynamics associated with underweight, overweight, and obesity, it is important to objectively assess energy intake (EI); energy intake assessment is related to food type recognition, amount consumed estimation, and portion size estimation [6]. Being underweight can result from energy expenditure exceeding energy intake over an extended period, which leads to health risks such as malnutrition and premature death [7]. Being overweight and obesity are associated with energy intake exceeding energy expenditure, leading to chronic diseases such as type 2 diabetes, cardiovascular diseases, cancers, and musculoskeletal disorders [6,7,8]. A dietary assessment system could be used to monitor daily food intake and control eating habits by triggering a just-in-time intervention during energy intake to prevent health issues [8].

Low-intake dehydration, caused by inadequate fluid intake, has endangered public health and is often underemphasised [9,10]. Mild dehydration happens commonly among people and increases the risk of chronic diseases [11,12]. A notable example is a significant association between urolithiasis (kidney stone) and low daily water intake [4,5]. Furthermore, dehydration is closely associated with disability, hospitalisation and mortality [13] in hospitals [14,15,16,17] and long-term care systems [10,11,13,18]. In the hydration and outcome in older patients (HOOP) prospective cohort study of 200 older adults in a large UK teaching hospital, 37% of the participants admitted as emergencies were dehydrated, 7% of the participants died in the hospital, and 79% of those who died were dehydrated at admission [15]. Dehydration and drinking status could also be related to children’s and adults’ attention and memory performance [19].

Existing literature reviews focus mainly on dietary monitoring and less on fluid intake monitoring. One representative review on fluid intake monitoring systems was [20], which grouped the methods by sensors, including wearable sensors, environmental sensors, containers/coasters, and surfaces [20]. However, this work only contains minimal narrative on vision-based methods. They did not investigate elements such as the camera types, placement of devices, and algorithms. In terms of dietary monitoring, Dalakleidi et al. (2022) and Wang et al. (2022) both presented reviews on vision-based food recognition/detection/segmentation and food volume estimation methods, namely vision-based dietary assessment (VBDA) [8,21]. Their work focused on processing RGB images and retrieving food features by training different deep convolutional neural networks (CNNs). At the same time, neither temporal information (e.g., video streams) nor information about other modalities (e.g., depth information) was considered. Complementing this, a review of the approaches to food intake detection was proposed [22]. Unlike VBDA, this review focused on detection methods of food intake movement, with both vision and non-vision sensors, in which camera and CNNs were still the dominating sensor and methodology [22].

Although both food and fluid intake are vital for human health and sharing similar body movements, including grasping (interacting with the container or food), delivering (bringing the object to mouth), and consuming (chewing or swallowing), there has been no review paper taking both food and fluid into consideration. Although ‘what’ (food/fluid detection/recognition/segmentation), ‘how much’ (quantity/volume estimation), and ‘when’ (intake action detection) are all crucial elements in intake monitoring, there have been few review papers addressing them comprehensively. Furthermore, the collaborative work between cameras and other non-vision sensors, frequently seen in the existing research, has not been adequately summarised. Consequently, we are providing this review paper that covers different monitoring approaches for food and fluid intake, targeting vision methods, sometimes applied with non-vision sensors such as inertial sensors, flex sensors, microphones, electromyography sensors (EMG), etc. The research gap and perspectives for future studies are identified, along with the hardware components, viewing angles, modalities of data, algorithms, and performance of the monitoring systems.

The rest of this paper is structured as follows: Section 2 details the methods of conducting the literature search and screening. Section 3 provides an overview of vision-based food and fluid intake monitoring methods, addressing the development of passive monitoring methods, and the awareness of privacy-preserving and free-living designs. Section 4 introduces the viewing angles and the devices, detailing the placement and usage of devices and data of different modalities. Section 5 casts insight into the tasks seen in intake monitoring and the algorithms. These tasks include binary classification (‘food/drink’ or ‘other’), food/drink type classification, intake action recognition, and food/fluid amount estimation. Section 6 is the discussion of the sections above. Section 7 gives a comprehensive conclusion of challenges and research gaps identified in the papers, providing perspective for future studies.

## 2. Methods

### 2.1. Literature Search

The databases that were employed include PubMed, SCOPUS, IEEE Xplore, ACM Digital Library, Web of Science, and Google Scholar. We selected search terms by combining the keywords and their synonyms. We set a weekly notification alarm for updates on new search results. Two stages were set for the search, presented as follows, where ‘+’ means ‘and’; ‘/’ means ‘or’:

Stage 1: The initial search terms included ‘vision/camera + fluid/drink/water/liquid/food/nutrition/energy/dietary + monitoring/detecting/recognition’; and ‘intake’ shall be found in the text. The advanced search function of the databases was used. Search terms were modified according to the rules of different databases, as shown in Table 1.

Stage 2: To extend the scope of the search, ‘vision/camera/image + human + action/gesture/activity/motion + recognition/detection/monitoring’ was searched across abstract and title. In addition, ‘drink/water/liquid/food/nutrition/energy/dietary’ shall be found in the text. This allows a wider and more exhaustive search to find potential papers involving intake monitoring in another research field (human action recognition, namely HAR).

### 2.2. Screening

The following eligibility criteria were applied: (1) at least one kind of vision-based technology (e.g., RGB-D camera or wearable camera) was used in the paper; (2) eating or drinking activities or both identified in the paper; (3) the paper used human participants data; (4) at least one of the evaluation criteria (e.g., F1-score) was used for assessing the performance of the design.

When the number of records in the search results exceeded 300, the first 100 were taken by the rank of relevance in each database, except for Google Scholar, in which only the first 50 records were taken. This is considering the enormous amount of data on Google Scholar, and most of the literature has been covered in other selected academic databases.

**Figure 1:**
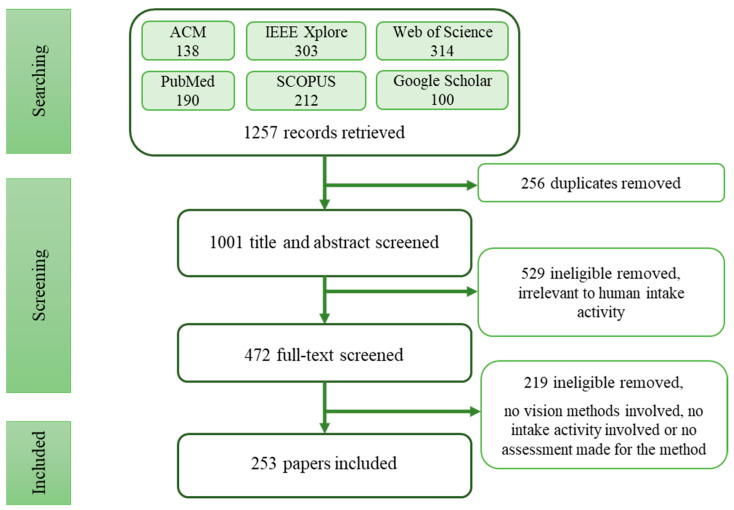
Diagram of paper searching and screening process. ACM is short for Association for Computing Machinery; IEEE is short for Institute of Electrical and Electronics Engineers; SCOPUS is a source-neutral abstract and citation database; PubMed is a free interface for searching MEDLINE, the National Library of Medicine’s premier bibliographic database, and the most popular bibliographic database in the health and medical sciences.

The retrieved records were first imported into Zotero, and duplicated items were removed. Then, all papers’ titles and abstracts were reviewed to remove articles not on human subjects; and those not mentioning visual methods in titles/abstracts. The full text review was applied in the next step of the eligibility assessment. Papers not mentioning intaking activities and not being evaluated with reasonable criteria were eliminated. Research on human action recognition and daily activity monitoring that addressed fluid intake activity were included. The screening process is summarised in the flow diagram of Figure 1, where 253 full texts from 2010 to 2022 were reviewed and included. There were 24 review papers, 34 proposing datasets, and 195 papers that provided methods, including algorithms, systems, or other solutions, for different tasks or problems for intake monitoring.

## 3. Overview of Vision-Based Intake Monitoring

### 3.1. Active and Passive Methods

In vision-based methods, there are two approaches to capturing images: active and passive [23,24]. Active methods require the user to take pictures and record their intake manually, while passive methods automatically access the food or fluid intake information. Active methods are widely used in practice. Traditionally, active food intake monitoring was in the form of food records, recalls, or questionnaires [25]. For active fluid intake monitoring, a fluid balance chart is used as a self-reporting tool to identify a positive (fluid input higher than output) or negative (fluid output higher than input) balance in hospitals or nursing homes [18,26]. A fluid balance chart includes information on the time, approach, and amount of body fluid input (oral, intravenous, etc.) and output (urine, tube, etc.), which can be completed by trained nurses, doctors, or patients themselves [26].

With the development of cameras, images of meals and drinks are more commonly used for dietary monitoring. In visual-based monitoring methods, active methods are not as widely seen as passive methods and mostly rely on mobile phone cameras. For example, [27] proposed a food and nutrition measurement approach by analysing the images taken by the users before and after a meal, which provided up to 97.2% of correct classification for food type and 1% of misreported nutrient information. A similar nutrition logger called DietCam was proposed in [28] based on self-taken videos or images before and after a meal for food type classification and intake amount estimation. Another food logger based on the active image captured using a mobile phone was developed with an inertial-smart watch and a load cell-smart [29], which also required manual food weighing. To reduce the time and efforts of human labour and improve the validity of portion size estimate, Jia et al. developed ‘eButton’, a semi-automated system which combined manual annotation with software and led to less bias and variability compared with manual annotation [30,31]. Another approach provided a user assistance system with a 360-degree RGB camera, combining active and passive methods to improve the quality of dietary and nutrition assessment. This system improved the solution of active food monitoring with fewer under-reporting cases and less perceived effort in keeping the food diary [24]. 

Nevertheless, manually recording the intake information by writing, weighing, or triggering a camera can be time-consuming and burdensome [24,32], hence not ideal for daily application. Moreover, self-reporting is not an option for patients with actional difficulties or the elderly with cognition degeneration. Therefore, in recent years, passive sensing methods with different devices and automatic strategies predominated over traditional active methods.

### 3.2. Environmental Settings

The environmental settings were categorised into the free-living environment, pseudo-free-living environment, laboratory environment, and others. In free-living-based studies, the systems were assessed with sufficient data collected by sensors configured into the user’s natural living environment. The pseudo-free-living environment tried to replicate the user’s natural living environment in a laboratory. The controlled laboratory environment only covers the specific actions needed as input data for the system (e.g., biting or drinking). In contrast, the others include methods based on existing datasets without considering the experimental environment. A camera’s viewing angle can be either first-person or third-person (details see Section 4). It is noticed that most of the third-person methods were only considered in a controlled testing environment, not testified in a free-living scenario. As for the first-person camera, in free-living, the highest accuracy achieved on food and non-food classification is 95% [33,34], and on eating action detection [35] was 89.68%. One recent free-living research reached the F1-score of 89% on drinking and eating episode detection but only 56.7% precision at the fluid intake level estimation [36]. Therefore, there is still a gap in harnessing cameras in the free-living scenario. Facts identified which can affect the performance include unstable light conditions, occlusion, low framerate, and motion blur.

### 3.3. Privacy Issue

Most papers investigating vision-based monitoring failed to discuss privacy issues even though some of the concerns were evident with the participant’s face and body shown in the figures in the paper [37,38,39,40,41,42]. In active methods, cameras can be controlled manually to avoid taking images with privacy concerns, which is inconvenient and labour-demanding [43,44]. Another approach seen in both active and passive methods was reviewing the photos after they were taken and deleting the ones with privacy concerns, which could also be time-consuming and burdensome [35,45]. Hence, passive methods with approaches to eliminate privacy issues were considered the most. Passive methods are more likely to face privacy concerns because most images captured passively are not related to food or drink consumption only [23]. Therefore, in some designs, the intake action was detected by a smart watch or glasses, and the camera was only turned on when the eating or drinking episode was highly probable [23,36,46]. In the survey of privacy concerns for users of AIM-2, the average level of concerns was reduced from 5.0 to 1.9 when images were captured only during intake action rather than continuously [23]. However, this method requires the users to wear multiple sensors, which can be cosmetically unpleasant, uncomfortable, and intrusive, especially for the elderly or groups with disabilities [44]. Moreover, the collaboration of different sensors increases the system’s complexity and makes it more challenging to maintain. An optional solution was detecting intake-related images from the cameras without the involvement of humans or extra sensors [44,47]. For example, a pre-trained MobileNet was used for food/non-food classification and helped the system save only food-related images [47]. However, this study’s precision was below 90%, so classification algorithms with better performance were needed. Additionally, the scenario that both the human face and food were presented in the same image was not mentioned, which remained to be considered. In other research, human face identification algorithms were applied to the obtained images to blur or remove the subjects’ faces [48,49,50]. Similarly, Android’s FaceDetector class was used in [33] to eliminate images with visible human faces. Some studies only relied on depth information from RGB-D cameras to reduce the concern of privacy [51,52,53,54], which could suffer from a high false positive rate [52], low accuracy (<90%) [54] or up to 148.8 mm error on mean distance [53]. Hence, algorithms with better performance tracking body movement and recognizing intake activities based only on depth information were needed. An alternative hardware solution to preserve privacy was proposed in [55], which creatively mounted the camera on a cap, facing down, to avoid capturing the surroundings.

## 4. Viewing Angles and Devices in Monitoring Systems

In passive vision-based intake monitoring, a camera’s viewing angle can be either first-person or third-person. A first-person camera (egocentric camera/wearable camera) is typically attached to the human body, pointing out at the food or container. In contrast, a third-person camera is mounted in the living environment, pointing at the subject. In the included papers that proposed a monitoring system or nutrition log application, 49 studies were based on first-person cameras, 39 on external third-person cameras, and 28 took advantage of the users’ smartphones. What is worth mentioning is that most of the phone-based methods were active, meaning the users needed to take the food/drink picture manually, e.g., the food/nutrition/dietary log proposed in [28,56,57]. This application based on mobile phones is not considered in the rest of this section, as we focus on passive methods and automatic systems.

There were four tasks identified in intake monitoring methods: binary classification, to distinguish food/drink intake from other activities; food/drink type classification, to detect the type of items consumed; food/fluid amount estimation, crucially related to energy intake; and intake action recognition, to recognize the human body movement. In the binary classification task, elements such as fingers, hands, containers, cutleries, and food can be detected, and different criteria can be set and followed as an indication of an intake activity.

**Figure 2:**
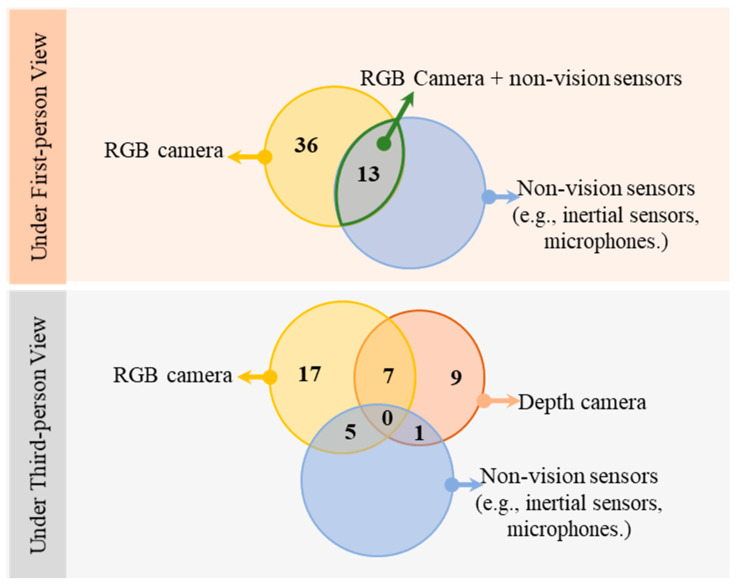
Pattern of the viewing angles and devices selected.

Regarding the placement of devices, first-person cameras could be in the form of glasses, watch and pendant, while third-person cameras could be mounted on the ceiling for a top-down view or placed around the subjects. The selected devices vary from studies, and cameras seen in the existing papers were mainly RGB and RGB-D cameras. RGB cameras have been spotted and used in combination with other non-vision sensors. The pattern of viewing angles and devices found in the papers is shown in Figure 2.

From the pattern of device selection shown in Figure 2, it is evident that the RGB cameras were the most used, primarily as first-person. In contrast, Depth cameras were not used as first-person cameras and were also barely used collaboratively with non-vision sensors. Moreover, there has been no system that covered all three sensors: RGB camera, Depth camera, and any of the non-vision sensors.

### 4.1. First-Person Approaches

As shown in Figure 2, of the 49 first-person methods, 36 relied on RGB information alone. The remaining 13 used RGB cameras collaboratively with other non-vision sensors, including accelerometers [23,50,58,59], gyroscopes, flex sensors [23,50], load cells [29], proximity sensors and IMUs [36]. The most common technology setting is an inertial-smart watch and a wearable RGB camera [33,34,46,55,60]. For example, Annapurna [33,34,60] is a smartwatch with a built-in camera proposed for autonomous food recording. The inertial sensor of the watch was used for gesture recognition to identify the eating activity, and then the camera took only pictures which were likely to be useful. Thus, compared to methods with a camera constantly in operation, redundant images were reduced, and so was storage requirement, privacy issue, computation, and camera power consumption. However, one fundamental problem with an inertial smartwatch is that the intake action could be missed when the user is drinking with a straw or using a hand that is not wearing the watch. Unlike the approaches mentioned above, which mainly focus on food intake detection was an intake monitoring system for fluid combining glasses, smartwatches, and phones [46]. The system achieved 85.6% accuracy on drinking action detection and 84% on liquids type classification.

Smart glass is another form of wearable device. Automatic Ingestion Monitor Version 2 (AIM-2) [23] was proposed with an accelerometer, a flex sensor and an RGB camera. However, in this design, images captured by the camera were only for validating the performance of other wearable sensors on intake detection; no visual methods were considered. FitByte [36] was a glasses-based diet monitoring system that applied six IMUs for chewing and swallowing detection, a proximity sensor for hand-to-mouth gesture detection, and an RGB camera pointing downward to capture potential food images. Both eating and drinking episodes were detected in this design. However, only 56.7% precision was achieved in fluid intake detection, while it was 92.8% for food intake. This means that Fitbyte performed significantly lower in fluid intake detection because it was only sensitive in simple and continuous drinking scenarios, other than short sips or when drinking happens with other irrelevant activities. A motion-adaptive algorithm was proposed for removing blurred images, which reduced the power consumption by 12% and increased battery life for the glasses-based system with an onboard camera and accelerometer [58].

Assessments have been made on the efficiency of first-person cameras for dietary monitoring. Thomaz et al. (2013) proposed and evaluated a dietary monitoring system based on a neck-worn and human computation. Images were taken by the camera every thirty seconds and sent on Amazon’s Mechanical Turk (AMT) (a platform providing human intelligence labour) for identifying food by human labour. This design resulted in 89.68% accuracy in identifying eating activities [35]. In 2015, the wearable camera SenseCam was used to evaluate its potential for dietary assessment. SenseCam made it possible to determine the subjects’ external environment, physical position, and interactive social condition. Regarding accuracy, only 71% of the eating episodes could be identified from the images. Hence, wearable cameras are deemed not reliable enough for individual application in dietary monitoring but acceptable as a complementary tool for enhancing traditional self-report [45,61].

The feasibility evaluations mentioned above revealed the limitations of utilising first-person cameras for passive dietary monitoring. What came first was the occlusion of view. For example, if the image did not provide a complete observation of food, the estimation accuracy of portion size could be low [30]. The uncertainty of wear time, battery sustainability and noncompliance in wearing the camera were other problems, especially when faced with the elderly or patients with cognition decline. As for image acquisition, dark and blurry images obtained in poor light conditions could make classification difficult. In addition, some of the eating episodes could be missed if they occur between shots, so a higher image-capturing frequency was needed. A higher frequency could raise the problem of a larger dataset, heavy computation, and substantial manual annotation. However, in recent research, wearable cameras were used to assess food and beverage consumption during transportation [43], which gave evidence that with the development of sensor applications and computer vision algorithms, the first-person camera can be used in dietary assessment in a free-living environment.

In summary, the common system architecture of first-person methods was combining one first-person RGB camera with other sensors. Cameras can be placed in the form of smartwatches [33,34], glasses [23,36], or even caps [55]. Combining cameras with other sensors can reduce the energy consumption of cameras, extend the use time of batteries, save storage space and rule out privacy concerns by turning the camera on only when a candidate movement is detected [33,34,60]. One fundamental limitation of inertial smartwatches is that the intake action could be missed when the user drinks with a straw or uses the contralateral hand with no watch. The incontinence of wearable devices was another limitation. Another fact worth noting is that in all the methods mentioned above based on RGB with non-vision sensors, the intake detection task was conducted by inertial sensors or proximity sensors rather than the camera itself. In other words, visual information was only used for food or fluid type classification and volume estimation instead of drinking action detection when used with non-vision sensors.

### 4.2. Third-Person Approaches

Compared to first-person cameras, third-person cameras have the advantage of being non-intrusive to the user’s [62]. The placement of cameras is one of the primary issues to consider. Most research had only one position of a single camera, placed on the ceiling for a top-down view [53,63,64], or placed pointing at the subject with a fixed distance from 0.6 m to 2 m [24,37,38,48,52]. Multiple cameras could be placed around for different viewing angles and complement the possible occlusion to achieve a more robust system [39,49,65]. However, there is no systematic comparison of the performance of single cameras or multiple cameras in various positions, so specific experiments are needed to choose suitable distances and pointing angles for dietary monitoring systems based on third-person methods.

In third-person methods, RGB and depth information can be used individually or collaboratively for action detection. Specifically, 17 papers used RGB information only, nine were with depth information from an RGB-D camera, and seven were based on the fusion of RGB and depth information, as seen in Figure 2. Unlike first-person cameras, non-vison sensors are used less frequently with third-person cameras. The main reason was that the kinematic information or distance information provided by IMUs and proximity sensors could also be obtained from the visual information of the third-person camera [37,52].

Microsoft Kinect was dominantly adopted in existing research, which can work day and night with the infrared sensor generating the depth images, and the skeleton tracking tool kit providing the joint coordinates [52,66]. The effectiveness of MS Kinect was tested for detecting the eating behaviour of older adults by placing the camera in front of the subject, resulting in an average of 89% success rate [37]. However, no occlusion problem was addressed, and only the experimental environment was considered in this research.

Regarding reducing privacy and image data concerns, some studies only used depth information from RGB-D cameras. For example, Kinect skeletal tracking was used for counting bites by tracking the jaw face point [67], and wrist roll joint of users based on depth information, achieving an overall accuracy of around 94% [52]. A system with an average accuracy of 96.2% was proposed, relying on the depth information of wrist joint and elbow joint motion obtained by a Kinect camera. However, although this study was presented for free-living calorie intake monitoring, only one camera position was tested, and no occlusion problem was considered [51]. The fusion of depth information and RGB information was another option, with the depth information for skeleton definition and body movement tracking while the RGB data for specific intake-related object detection [63].

RGB cameras were also popular devices in intake monitoring used as third-person cameras. It can be embedded in the ceiling, pointing down [64], or put on the dining table, pointing at the subject [38]. The fusion of RGB and depth information has the potential to reach higher accuracy compared to using a single modality of information. An example can be seen in [62], where an adapted version of the self-organized map algorithm was applied to the skeleton model obtained from depth information for movement tracking. The RGB stream was for recognising eating-related items such as glass. This method achieved a 98.3% overall accuracy. All RGB-D cameras were used as third-person cameras (as seen in Figure 2). Table 2 details the methods and accuracy of the seven papers utilising both RGB and depth information, which indicated that the collaborative use of RGB and depth information has become popular in recent years and has the potential to provide promising performance in intake monitoring tasks.

## 5. Algorithms by Task

As was mentioned in the introduction, four tasks were observed in the papers. In these four tasks, binary classification (‘food/drink’ versus ‘other’), food/drink type classification and food/fluid amount estimation mainly focused on retrieving information from the image of food/drink, while intake action recognition was aimed at human body movement.

**Figure 3:**
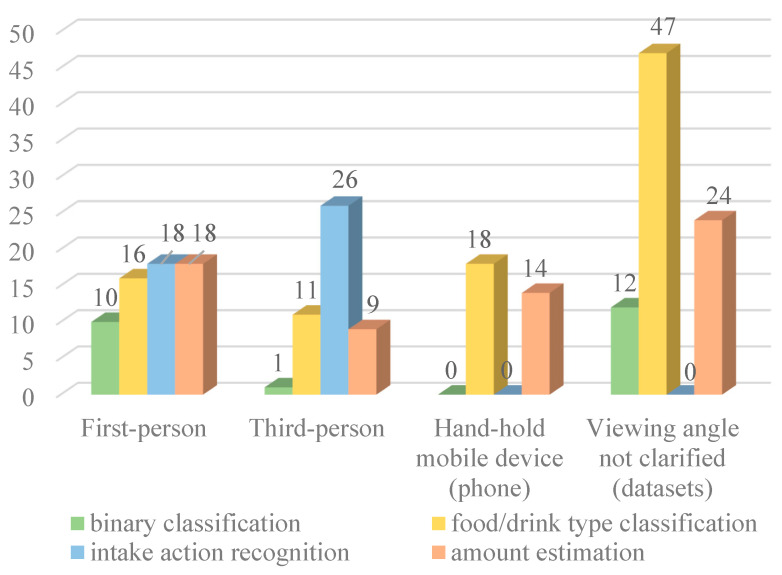
Tasks of the vision-based intake monitoring methods with viewing angles.

The viewing angles and tasks are illustrated in Figure 3, indicating that third-person cameras are mostly used for intake action detection but were not commonly used for food/drink classification tasks or amount estimation. On the contrary, first-person cameras are commonly used for food/drink detection or amount estimation rather than action recognition. The algorithms used in each task are shown in the following subsections.

**Figure 4:**
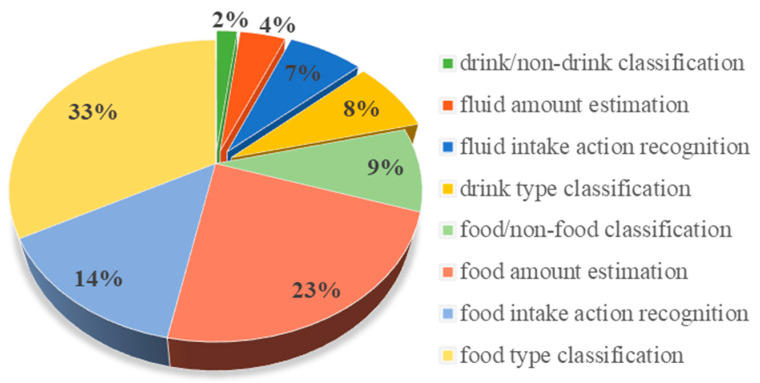
Pie chart of paper allocation on different tasks. Darker colour indicates methods that consider fluid intake, while lighter colour indicates those considering food intake.

The proportion of papers on these four tasks is demonstrated in Figure 4. The pie chart shows that food research has been dominantly outnumbering fluid. Moreover, the food and fluid type classification were the most attended intake monitoring task, followed by the amount estimation task. However, the efforts on drink/non-drink classification and fluid amount estimation were significantly limited.

### 5.1. Binary Classification

Eliminating unrelated images was a preliminary step for identifying candidate intake activities. This was commonly proposed as a binary classification approach to distinguish food/drink from other objects or to classify low-quality images and delete them. For example, to distinguish sharp images from blurry images for adequate image quality, Fast-Fourier Transform (FFT) for images was calculated to analyse the sharpness, resulting in a 10–15% misclassification [50].

Im2 Calories was a food intake monitoring system proposed in 2015, where a GoogLeNet CNN was trained with a modified Food101 dataset. One of the tasks for Im2 Calories was to determine whether the image was related to a meal, at which an accuracy of 99.02% was achieved [73]. Similarly, a GoogLeNet model was trained for food/non-food classification by Singla et al. [74] and achieved an accuracy of 99.2%, in which Food-5K created from Food101 was used as training data. The two works mentioned above were based on the same pre-trained model and similar food dataset and both had promising performance on the binary classification task. Another example was iLog, a stress-eating monitoring system based on a seven-layer CNN model and camera-mounted glasses, which achieved around 97% accuracy in food detection [75].

The GoogLeNet in Im2 Calories [73] was tuned on a Titan X GPU with 12 GB memory; then implemented into an Android APP less than 40 MB, which could classify an image within one second. iLog could also operate on edge-level, low-performance computing paradigms, such as mobile phones, sensors and single-board computers [75]. Apart from the networks mentioned above, for real-time and portable monitoring, a derived MobileNet was proposed and implemented into a Cortex-M7 microcontroller for dietary image capturing, which achieved an average precision of 82% in identifying food-related images [47]. The training was conducted on Google Colab using 400 food images and 400 non-food images, taking up to 5.5 h, while only 761.99 KB of flash and 501.76 KB of RAM were needed to implement this algorithm. Hence, networks such as GoogLeNet and MobileNet could provide portable, edge-level, and real-time solutions for binary classification tasks. Still, the training process could be demanding on computation power, where a high computing performance device/server was needed.

Annapurna was a multimodel system with a camera mounted on an inertial smartwatch for dietary recording [33,34]. In this design, the camera was only switched on when the watch detected intake action. A mobile phone was first used as a lightweight computing platform to eliminate images with human faces and blurred edges. Then, 37% of the remaining images with food items in them were transferred to a server for further processing, where the Clarifai API was used to identify the presence of food items in pictures based on CNN, and a depth map was created to detect food too far from the camera (considered as unrelated to the meal). As a result, 95% of the meals could be recalled by the proposed system in a free-living environment. The computation was firstly on mobile phones for Annapurna to remove blank, blurry, and misleading images to reduce runtime for further computing. However, the latency was around 0.9 s for the smartwatch to capture an image, which limited the response speed of the whole system [34].

The server used in Annapurna [33,34], Clarifai API, was also used in [44], where it generated tag outputs (e.g., ‘food’, ‘car’, ‘dish’.) of an input image for determining whether the image was food-related. This method was tested on both Food-5K and e-Button and reached the specificity of 87% on Food-5K (created in [74]), higher than the results on e-Button. This was because e-Button was an egocentric free-living dataset with 17.7% blurred images, complex backgrounds, and more diverse objects. According to the authors, although the burden of manually observing and recording dietary activities in previous work [76] was reduced, the effectiveness of automatic monitoring was still limited due to the quality of the captured images.

Only limited papers addressed the binary classification of fluid/drink/beverage. An example covering food and fluid was [77], which trained a YOLOv5 network to detect and localize food and beverage items from other objects. The study aimed to distinguish food and beverages from other objects and added ‘screen’ and ‘person’ as extra classes. As a result, an overall mean average precision of 80.6% was achieved for classifying these four objects, which was still far from being used in practice. NutriNet was another deep Neural Network proposed for both food and beverage; the detection model’s output was either ‘food/drink’ or ‘other’ [78]. NutriNet was trained using the NVIDIA GeForce GTX TITAN X in a local computer and fine-tuned on an NVIDIA Tesla K80 in a server environment. This was compared to AlexNet, GoogLeNet and ResNet with three different solver types (SGD, NAG and AdaGrad), in which the NutriNet with the NAG solver achieved the best detection accuracy of 94.47%. Among the compared networks, the training time could take up to 135 h, whereas the ResNet models were the most time-consuming due to the deep learning architecture.

It is noted from the above review that most of the binary classification tasks were based on first-person images. Obtaining a clear and intake-related image is the preliminary step in vision-related intake monitoring technologies. Deep learning algorithms were used for detecting food/drink-related images and eliminating irrelevant images, which achieved promising results (up to 99.2%) on food-related datasets such as Food101 and Food-5K. However, according to the e-Button dataset result, developing robust algorithms for free-living data are still a challenge because the body movements could easily cause blur and occlusion to the captured image. The existing methods have focused on food/non-food classification, while only a few address both food and beverages.

### 5.2. Food/Drink Type Classification

After images of interest were acquired, more advanced classification was needed to identify the intaking food/drink type. Table 3 provide an organized overview of the algorithms used in different papers. In the investigated papers, 29 adopted machine learning (ML) methods; 51 used deep learning (DL) methods, and 11 conducted other methods.

ML methods included support vector machines (SVM), principal component analysis (PCA) [69,75,79], K-means classifiers, random forest (RF), fully connected neural networks (NN), artificial neural networks (ANN) [80], and some image matching and retrieving methods such as content-based image retrieval (CBIR) [81], dynamic time wrapping (DTW) [82], bag of features (BoF) [40,83,84], which clusters the features into visual words. Features of the image could be extracted by methods including speeded-up robust features(SURF) and scale-invariant feature transform (SIFT). In these methods, SVM was most seen and often used collaboratively with other DL methods. Different networks were seen in DL methods, such as GoogLeNet [73,74,78,80,82,85,86,87,88], MobileNetV2 [69,79], AlexNet [78,85,86], Inception-V3 [82,88,89,90,91], NutriNet [78,86,92], K-foodNet, and Very deep convolutional neural network [85], DenseNet161 [82], fully convolutional networks (FCN) [86,92,93,94], YOLO [93,95,96], extreme learning machine (ELM) extreme [97,98,99], neural trees [97], graph convolutional networks(GCN) [100], deep learning PDE model (DPM) [83], SibNet [101], VGG16 or VGG365 [82,85,94,102], ResNet, ResNet50 and ResNet152 [78,80,82,85,86,88,89,90,103,104], EfficientNet [105], EfficientDet [106], faster-RCNN [46,90,104]. GoogLeNet and ResNet, with their variant, were the most popular. Except for learning-based methods, other methods, including the region growing algorithm [69], mean-shift [40] algorithm, template matching [107,108], and other image processing algorithms were also used for image segmentation, recognition and even amount estimation.

Similar to Section 5.1 for the binary classification task, most of the food/drink type classification approaches were based on first-person cameras, and most of them were only for food type identification, with beverages not included. An example of drink type classification was [40], in which the method of drink region segmentation and the development of a bag of features (BoF) was proposed. Both speed-up robust features(SURF) and colour-based features were used for recognizing the types of drinks, and an accuracy greater than 89% was achieved [40]. HydraDocter was another example of fluid intake [46], where a trained faster-RCNN was used for container identification and classification from the captured videos. In this work, six types of drinks, including juice, coffee, cola, water, milk, and beer, were classified of which coffee and milk achieved the best accuracy, and the overall accuracy was 84.3%. The challenge was that poor image quality caused by the position and viewing angle of the container in the image made recognition difficult. As a solution, HydraDoctor captured a set of images (a short video) to decide the drinking period, and the validated images were taken after the drinking action was completed [46]. However, although this study was providing a real-time monitoring system, the runtime of the algorithm was not provided in the paper.

An early study harnessed SVM with a Gaussian radial basis kernel for training a classifier on food type achieved an accuracy of 97.3% when the training data took up to 50% of the dataset [27]. Surprisingly, only 1% of misreported nutrient information was found in this study. However, there was only one food item in each image, so the robustness of the proposed algorithm could be limited when tested on images with multiple food items or complex backgrounds [27].

As mentioned in Section 3.1, DietCam was a food logger depending on self-taken images, which resulted in 92% accuracy in the food classification [28]. The food was first recognised by matching it with a food database, and three images of one item were required to reduce the risk of occlusion. OCR techniques and user input were optional for food that was not differentiable by appearance. Disappointingly, the matching algorithms used were not suitable for classification (for example, ‘cheeseburgers’ and ‘double cheeseburgers’ were the same type of food with different appearances), so a Bayes decision theory-based probabilistic algorithm was proposed for food classification after matching [28]. Another observation was that the accuracy was positively related to the number of references in the database. Hence an extensive database was needed for a large number of patterns covered, leading to high accuracy [28].

For automatic and larger-scale image analysis, computer vision algorithms were then used in later research. CNNs trained by labelled image data provided another method for food classification. Im2Calories mentioned in the last section were examples of a GoogLeNet CNN being trained with different datasets created from existing datasets online. Im2 Calories [73] trained the GoogLeNet with a self-made multi-label dataset and achieved an average precision of 80% [73]. In Ref. [74], Food-11 was created for training, validation, and evaluation, resulting in recognition by 83.6% of food categories. The work mentioned in Section 5.1 verified the performance of CNNs on food/non-food classification tasks. However, the accuracy of food type recognition was limited. The reason could be the mixture of food items in images and the similarity across some food categories. Hence, to achieve higher accuracy on food type recognition, multiple training labels and multiple outputs for one image were suggested for further research, so as different CNN models [74]. NutriNet was also trained for food and beverage recognition with an accuracy of 92.18% [92]. This experiment was conducted on a server equipped with an Intel Core i7-8700K CPU, an Nvidia GeForce GTX 1080 Ti GPU, and 32 GB of RAM. However, NutriNet was limited to only one output for each image, so pixel-level classification was then considered for recognizing multiple food and beverages in one image [92]. To be specific, the FCN-8s Network was used to output a pixel map instead of a single result [109].

Deep neural networks were most likely to achieve extremely high performance (over 99% accuracy) in the classification and recognition tasks. The networks could be used for both food and fluid classification in which Inception ResNet V2, ResNet50 V2, ResNet152, MobileNet V2 and V3 and GoogleNet were seen with over 95% accuracy. Apart from deep neural networks, machine learning methods such as RF, SVM, KNN etc, could also reach over 90% accuracy. However, DL methods could require high-performance devices and be time-consuming when training and the performance of models rely on a sufficient amount of training data with variety. The value of a deep neural network lies in the trade-off between its performance and simplicity.

**Table 3:** Summary of the algorithms used in the classification task with viewing angles.

(a) Part 1										
References		Methods			Details of the Methods	Assessment	Drinking Included (Y/N)	Viewing Angle Applied		
		ML	DL	Other				First-Person	Third-Person	Not Mentioned
Qiu et al., 2021	[110]		1		DNN	Bite counting: 74.15% top-1 accuracy and an MSE value of 0.312 when using regression.	Y	1		
Martinez et al., 2020	[102]	1	1		Compared with, VGG365, FV (fine-tunning VGG365) + RF, SVMTree, FV + SVM, FV + KNN, MACNet, EnsembleCNN.	Food classification: an accuracy and F-score of 56% and 65% on the EgoFoodPlaces dataset.	Y	1		
Du et al., 2019	[46]	1	1		SVM on the phone for hand raising action; Faster-RCNN on the server to confirm the detected drinking activity and identify the type of drink.	Drinking activity detection was 85.64%, and types of liquids classification was 84%, respectively.	Y	1		
Fuchs et al., 2020	[89]		1		CNN (Inception ResNet V2, ResNet50 V2, and MobileNet V2) for beverage recognition.	A mAP of over 95% was observed when 100 images per product were used for training.	Y	1		
Zhu et al., 2010	[27]	1			Take food pic before and after eating; SVM with a Gaussian radial basis kernel for classification; camera parameter estimation and model reconstruction for volume estimation.	Up to 97.2% for classification and 1% misreported on Nutrition Information (with 50% training data).	Y		1	
Zhang et al., 2018	[69]	1	1	1	Segmentation of point cloud on colour and depth information using region growing algorithm; CNN for recognition and PCA for orientation estimation.	Average of 90.3% success rate when detecting and estimating the orientation of handle-less cups.	Y		1	
Bellandi et al., 2012	[108]			1	Halcon “Matching Assistant” tool: template matching.	Available for both object detection and Locate Glasses procedure.	Y		1	
Sadeq et al., 2018	[111]		1		CNN.	96% on food recognition on the FooDD dataset.	Y		1	
Rouast et al., 2018	[24]		1		CNN.	70% accuracy on action classification with class-balanced test data.	Y		1	
Chae et al., 2011	[107]	1		1	Medial axis to determine the potential width of the template shape (shape boundary); the active contour methodology.	For 17 beverage images average relative error and standard deviation were about 11% and eight, respectively. For bread slices, the volume was 8% overestimated.	Y		1	
Hafiz et al., 2016	[40]	1		1	Colour-based BoF; Speed up robust features (SURF); Mean-shift segmentation.	>89% accuracy.	Y			1
Park et al., 2019	[112]	1			A hierarchical multi-task learning framework.	Sugar level prediction with over 85% accuracy and alcoholic drink recognition over 90% accuracy.	Y			1
Mezgec et al., 2017	[78]		1		A new architecture; Compared with four different deep learning architectures (AlexNet, GoogLeNet, ResNet and NutriNet) and three solver types (SGD, NAG, and AdaGrad).	A classification accuracy of 86.72%, a detection accuracy of 94.47% (the binary task).	Y			1
Mezgec et al., 2019	[92]		1		DL-FCN-8s network, namely NutriNet.	Accuracy of 92.18%.	Y			1
Mezgec et al., 2021	[86]		1		DNN, namely NutriNet for food recognition (tested against AlexNet, GoogLeNet and ResNet); FCNs and ResNet for food segmentation.	AP up to 63.4% on food recognition (result from food recognition challenge).	Y			1
Schiboni et al., 2018	[55]		1	1	Image Processing and SVM for recognition and basic mathematical equation for calculating calories.	90% recall on food detection.	N	1		
Liu et al., 2018	[113]			1	Food segmentation by an improved C-V model; 3D virtual object construction.	MAPE values for food volumes measuring were mostly below 7%; average MAPE values for all objects acquired were 4.48%.	N	1		
Zhou et al., 2022	[114]	1			Cross-model retrieval on diabetogenic food (CMRDF). A graph-based cross-modal retrieval method.	The mAP is up to 75.9% using CMRDF-3M.	N	1		
Qiu, Lo, Gu, et al., 2021	[104]		1		Transformer-based captioning model (Faster RCNN + ResNet were in use); Compared to other 4 algorithms.	42.0 on Volume Estimation, 47.5 on Food Recognition and 62.7 on Action Recognition.	N	1		
Jiang et al., 2018	[81]	1			AR overlay application: object tracking based on content-based image retrieval (CBIR) by RIS (a form of content-based image retrieval).	The average recognition rate is 75.9% at the supermarket Scene and 87.9% on FIDS30 Database.	N	1		
Rachakonda et al., 2020	[75]	1			Machine learning for food classification; Principal Component Analysis (PCA).	97% total accuracy for object detection (not for energy estimation or action detection).	N	1		
Rachakonda et al., 2019	[115]		1		Tensorflow and object detection interface; Firebase Database for nutrition estimation.	97% accuracy for calorie counts.	N	1		
Matei et al., 2021	[82]	1	1	1	CNN for food and place detection (ResNet50, DenseNet161, and VGG16 combined with SVM, KNN, RF for binary classification); Object detector: ResNet50, DenseNet161, VGG16, GoogLeNet Inception V3 architectures pre-trained on ImageNet; Place recognition: ResNet50 pre-trained on Places365; DTW for nutritional activities analysis, output the similarity of two-time series; Isolation Forest for grouping days with same nutritional habits.	A weighted accuracy and F-score of 70% and 63%, respectively, on food/non-food classification.	N	1		
Konstantakopoulos et al., 2021	[116]			1	Two-view 3D food reconstruction.	A mean absolute percentage error from 4.6–11.1% per food dish.	N		1	
Rahmana et al., 2012	[117]			1	Generating texture features from food images using GABOR filters.	MAP values remain above 90% for the extreme scale factor values of 0.7 and 1.4. Tables on food classification and image retrieval	N		1	
Iizuka et al., 2018	[68]		1		NN and CNN.	Estimate the weight of each meal element within a 10% error rate.	N		1	
Qiu et al., 2019	[118]		1	1	Mask R-CNN + a mechanism for hand-face distance.	See Table 2 in the literature and the description.	N		1	
Lei et al., 2021	[119]		1		Mask R-CNN for dish detection + OpenPose for pose estimation (set threshold for the 75 values obtained).	Eating state estimation 87.7% average accuracy; bite error percentage 26.2%.	N		1	
Sarapisto et al., 2022	[103]		1		ResNet for food recognition, deeper ResNet with MenuProd for wight estimation.	90% F1 score (averaged over classes) in a multi-label classification task of detecting the food items; approximately 15 g error per food item over all items.	N		1	
Rhyner et al., 2016	[120]				Assessing the accuracy of GoCARB APP.	85.1% successfully recognised by the APP.	N		1	
**(b) Part 2**										
Esfahani et al., 2020	[121]	1			SVM (Support Vector Machine) and Logistic Regression classifiers.	Classification accuracy 0.5119 on hyperspectral and 0.4558 on RGB using SVM.	N		1	
Kong et al., 2011	[122]	1			SVM.	76% and 84% when recognizing arbitrary number of or single food item respectively.	N		1	
Tomescu, 2020	[105]		1		EfficientNet for food recognition and depth map fusion for estimating volume.	A slight volume overestimation of 0–10%.	N		1	
Myers et al., 2015	[73]		1		A CNN-based classifier (GoogleNet).	99.02% on meal detection; an average relative error of 0.18 m on volume estimation.	N		1	
Pouladzadeh et al., 2013	[123]	1			K-mean-Clustering for features; SVM.	92.2% accuracy for food recognition (with all features used); area measurement 6.39% error on average. no results on calories estimation.	N		1	
Jayakumar et al., 2020	[124]		1		CNN for food and face detection.	Accuracy of food detection was not clear but for face detection was a table.	N		1	
Lee et al., 2016	[79]	1			Extract HOG features from IR and RGB then SVM +PCA + kPCA.	Isolate food parts with an accuracy of 97.5% and determine the type of food with an accuracy of 88.93%.	N		1	
Gao et al., 2019	[94]		1	1	Maximum Length Sequence (MLS) in sound signal and single-task FCN (VGG-16) for image; (neither training images with volume information nor placing a reference object of known size).	Relative error of −0.27% to 12.37% on different objects.	N		1	
Ravì et al., 2015	[125]	1			A Fischer Vector representation together with a set of linear classifiers are used to categorize food intake based on color and texture; then being implemented with an activity recognition APP.	0.73–0.78 of classification rate on Top-5 candidates on UEC-FOOD100; recognition rate over 84% for the 6 activities.	N		1	
Zhu et al., 2010	[126]	1			Image analysis (CIELAB color space) and SVM for classification; 3D reconstruction for estimation on volume.	Mean classification accuracy 95.8% when 50% training data; error rate on estimation 3.4–7.0% for large item but 36.6–56.4% for small items.	N		1	
Singla et al., 2016	[74]		1		GoogLeNet.	99.2% on food/non-food classification; 83.6% on food categorization.	N			1
Anthimopoulos et al., 2014	[84]	1			Bag-of-features (BoF) model.	Classification accuracy of the order of 78%.	N			1
Khan et al., 2019	[127]		1		CNN.	Accuracy of 90.47%.	N			1
Lu et al., 2018	[128]		1		CNN for food segmentation, recognition, depth prediction and volume estimation.	Significant improvement on all results comparing to another paper ‘A Multimedia Database for Automatic Meal Assessment System’ (See tables in literature).	N			1
Almaghrabi et al., 2012	[129]	1			Image Processing and SVM for recognition and basic mathematical equation for calculating calories.	89% accuracy for food recognition using SVM. 4.22% error for calories estimation.	N			1
Pouladzadeh et al., 2012	[130]	1			SVM.	92.6% on food categories recognition.	N			1
Martinel et al., 2015	[98]	1	1		Extreme Learning Machine + SVM to select features.	Score improvement up to + 28.98; score (accuracy of the proposed method).	N			1
Martinel et al., 2016	[99]	1			Extreme Learning Machine specialize a single feature type (e.g., color); structured SVM for feature filtering.	Significant higher accuracy on different datasets for food classification.	N			1
Ruenin et al., 2020	[90]		1		1. Faster R-CNN and select ResNet-50 as a pre-trained model; 2. CNN which uses a a pre-trained model as InceptionResNetV2.	mAP = 73.354 for first part; MAPE = 16.9729 for the second part	N			1
Islam et al., 2018	[131]		1		1. Transfer learning and re-train the DCNNs on food images; 2. extract features from pre-trained DCNN to train classifiers.	Up to 99.4% on Food-5K.	N			1
Pfisterer et al., 2022	[132]		1		Deep convolutional encoder-decoder food network with depth-refinement (EDFN-D).	IOU: EDFN-D 0.879; Depth-refined graph cut 0.887. Intake errors well below typical 50% (mean percent intake error: −4.2%).	N			1
Chen et al., 2017	[133]		1		Bilinear CNN models.	84.92–99.28% classification rate on UECFOOD-100 and UECFOOD-256 dataset.	N			1
Tammachat et al., 2014	[134]	1			SVM.	Overall accuracy 70% on food type recognition and 50% on calorie estimation.	N			1
McAllister et al., 2018	[80]	1	1		Pre-trained ResNet-152 and GoogleNet CNN for feature extraction + classification based on machine leaning using ANN, SVM, Random Rorest, fully connected NN, Naïve Bayes.	ResNet-152 deep features with SVM with RBF kernel can accurately detect food items with 99.4% accuracy using Food-5K food image dataset.	N			1
Liu et al., 2022	[106]		1		EfficientDet deep learning (DL) model.	mAP = 0.92 considering 87 types of dishes.	N			1
Liu et al., 2016	[135]		1		CNN.	Top-5 Accuracy 94.8% on UEC-100 and 87.2% on UEC-256.	N			1
**(c) Part 3**										
Li et al., 2022	[96]		1		YOLOv5 for food recognition.	89.7% for food recognition; 90.1% for average nutrition composition perception accuracy.	N			1
Tahir et al., 2021	[136]		1		MobiletNetV3 with weights from a pre-trained model of ImageNet.	99.1 F1 score on food/non-food classification; 81.46–91.93% Top3 food recognition F1-score.	N			1
Martinel et al., 2018	[137]		1		DNN, a new architecture using a slice convolution block to capture the specific vertical food traits; tested on UECFood100, UECFood256 and Food-101.	A top–1 accuracy of 90.27% on the Food-101 dataset.	N			1
Christodoulidis et al., 2015	[138]		1		6-layer deep CNN.	Overall accuracy of 84.9%.	N			1
Yang et al., 2010	[139]	1			Get pairwise features on 8 different ingredient types; feature vector for discriminative classifier: Baseline algorithms: colour histogram + SVM and bag of SIFT features + SVM.	Nearly 80% with OM (a joint feature of orientation and midpoint).	N			1
Miyano et al., 2012	[140]	1			Bag-of-Features representation using local descriptors and color feature; Histogram intersection approach and SVM as classifier.	With both BOF and color feature, HI achieved 89.3% and SVM 98.7%.	N			1
Zhao et al., 2021	[100]		1		Graph Convolutional Network (GCN) to learn inter-class relations.	See table of different feature extractor, and different methods (1-shots, few-shot, fusion) on Food-101 and UECFood-256.	N			1
Lu et al., 2020	[91]		1		DNN (Inception-V3 for food recognition).	For food recognition: highest 78.2% Top-3 on Hyper2-MADiMa database; see tables for food segmentation and nutrition estimation.	N			1
Aguilar et al., 2018	[93]		1		FCN (Tiramisu model) for food segmentation, Yolov2 for food detection.	90% F-measure on UNIMIB2016.	N			1
Bettadapura et al., 2015	[141]	1			Geo-Localizing Images; image processing (Color Moment Invariants, Hue Histograms, C-SIFT, OpponentSIFT, RGB-SIFT, SIFT); weekly Supervised Learning to train SMO-MKL multi-class SVM classification framework.	Average performance increased by 47.66% when location prior was included. (15.67%. to 63.33%).	N			1
Martinel et al., 2016	[97]		1		CNN, Extreme Learning Machines (ELM) and Neural Trees.	69.3% (significantly improved from 60.2% (PMTS [paper “Real-Time Photo Mining from the Twitter Stream: Event Photo Discovery and Food Photo Detection”]) to 69.3%.).	N			1
Zhu et al., 2011	[142]	1			Salient Region Detection based on color and multiscale segmentation by SVM.	Average classification accuracy for 32 food classes is 44%.	N			1
Yumang et al., 2021	[95]		1		YOLO. trained with Pyimagesearch on classification and a formular for distance calculation.	The accuracy of the device to be 0.77 or 77% on classification and a small discrepancy of almost 0.1–0.9 margin on distance estimation.	N			1
Wang et al., 2015	[143]	1	1		Bag-of-Words Histogram (BoW) + SIFT for vision; Bossanova Image Pooling Representation; deep CNN and very deep CNN.	The fusion of visual and textual information achieves better average precision 85.1%.	N			1
Teng et al., 2019	[144]		1		A 5-layer deep CNN.	Top-1 accuracy of 97.12% and the top-5 accuracy of 99.86%.	N			1
Matsuda et al., 2012	[83]	1	1		Candidate regions detection by DPM (a Neural Network), a circle detector and the JSEG region segmentation; feature-fusion-based food recognition method using features including bag-of-features of SIFT and CSIFT with spatial pyramid (SP-BoF), histogram of oriented gradient (HoG), and Gabor texture features.	55.8% classification rate for a multiple food dataset.	N			1
Poply et al., 2021	[145]		1		CNN for object detection and semantic segmentation.	mAP of 89.3% of object detection, percentage accuracy of 93.06% for calorie prediction.	N			1
Nguyen et al., 2022	[101]		1		Deep CNN namely ‘SibNet’.	MAE 0.13–0.15 for counting; PQ (panoptic quality) 81.68–89.83% for segmentation.	N			1
Ege et al., 2017	[146]		1		Multi-task CNN.	Average classification accuracy on the top-200 samples with the larger error was 71%, with the smaller error 86%; Table 3: The results on calorie and category estimation.	N			1
Bolaños et al., 2016	[87]		1		CNN + a Global Average Pooling (GAP) layer + Food Activation Maps (FAM) (food heat map) on food detection (food/non-food), namely GoogleNet-GAP.	Validation accuracy up to 95.64% on food/non-food; up to 91.5% on food recognition; on simultaneous test on localization and recognition see Table 2 of mean accuracy.	N			1
Ciocca et al., 2020	[88]		1		CNN (GoogLeNet, Inception-v3, MobileNet-v2, and ResNet50) + SVM; combined deep-based and hand-craft features.	Details are in tables of the paper, Inc-V3 + LBP-RI + SVM (RBF) achieved the best performance.	N			1
Park et al., 2019	[85]		1		DCNN (namely K-foodNet), compared with AlexNet, GoogLeNet, Very Deep Convolutional Neural Network, VGG and ResNet.	Test accuracy 91.3% and recognition time 0.4 ms.	N			1

### 5.3. Intake Action Recognition

The process of an intake activity can be segmented into preparing, delivering, and swallowing, where the preparing phase includes the action of grasping a container and delivering refers to lifting hands to one’s mouth. Most of the methods took the observation of food or fluid in human hands as a representation of intake, which turned the action recognition problem into a simple object detection problem. However, taking the presence of food/drink objects as the representation of intake activities has a high false positive rate. For example, in [44], some food preparation and shopping images were misclassified as intake-related images. Hence, identifying the actual body movement of intaking was optimal and more challenging. Efforts have been made to recognise body movement and understand human behaviour through vision. In terms of intake monitoring, the last section mainly focused on the ‘what’ problem, trying to monitor what the person was drinking or eating, while this section will be about ‘when’.

Most of the action detection tasks depended on third-person cameras rather than first-person cameras; in those third-person cameras, depth cameras were more popular than RGB cameras. Microsoft Kinect was the most used device, of which the SDK could provide skeleton tracking for 25 joints on each body for up to six people, from 0.8 to 4 m, as well as six types of streams, including depth, infrared, colour, skeleton and audio [52,53]. As for hardware settings, [53] was tested on a computer running Windows 8 with an Intel Core i5 processor and 8 GB of RAM.

Staring with third-person methods, RGB information was first used for intake detection before the development of the depth camera. One example was the method based on fuzzy vector quantization proposed in 2012, in which activities were considered as 3D volumes formed by a sequence of human poses [48]. Fuzzy vector quantization was for associating the 3D volume representation of an activity video with 3D volume prototypes; the linear discriminant analysis was then used to map activity representations in a low-dimensional discriminant feature space. In this space, a simple nearest centroid classification procedure was used to classify activities, including eating, drinking and apraxia, which achieved an overall correct classification rate of 93.3% [48]. There was no mention of the computational requirements, or the hardware used for the experiments.

Another example using only RGB information was a real-time eating monitoring system for Alzheimer’s patients presented in 2018 [38]. This design detected human hand movements, with an RGB camera pointing at the subject, resulting in 89% accuracy with a frame rate of 3.9 fps. In this study, hand and mouth regions were detected by the Haar-Cascade classifier, and the HSV skin-colour filtering approach was used for tracking the hand movements between two reference points, of which one was the position of the mouth, and another was a referencing object put by the food tray. The notable limitation was that an extra reference point was needed near the food, which complicated the system [38]. With the development of deep learning and CNNs, an automatic eating monitoring system was proposed by firstly identifying faces using a Faster R-CNN and then counting bites and chews from affine optical flow parameters using a pre-trained AlexNet on MATLAB [39], which achieved an accuracy of 85.4% ± 6.2% in counting bites and 88.9% ± 7.4% in counting chews. False prediction in this research was mainly caused by gestures resembling bringing hands to the mouth, such as wiping the mouth [39].

The Naive Bayes classifier was first used with Kinect in [3] in 2013 to classify the input images for patient fluid intake monitoring. The performance with different positions of the subject and partial occlusions of the camera was tested. However, the limitation found in this method was that Naive Bayes classifier was only applicable to a relatively small dataset and test case, so the effectiveness of this approach in large-scale free-living scenarios remained to be validated. Moreover, the experimental test set for the method was insufficient, with only three replications and 10 s data for each [3]. The Naive Bayes classifier was used because it assumes each attribute is mathematically independent and can find prior probabilities with small datasets.

In 2014, an automatic drinking activities identification system was proposed based on dynamic time wrapping (DTW) algorithm [54]. DTW is an algorithm that computes the distance between two different signals and analyses the final cost distance to identify the differences between the signals [147], commonly used in speech recognition and ECG signal recognition. The distance between the user’s hands and the camera was used to judge whether the person was drinking. A total accuracy of 89% was achieved when being tested with three camera locations [54].

In later years, more information from the camera, rather than a single indicator, was used for more accurate detection. In [37], the skeleton coordinates from the depth image provided by Kinect were used for analysing the movements of drinking soup, drinking water, and eating the main course. The distance from both hands to the head and the plate to the head were used as characterisation for classifying the gestures, resulting in an 89% average success rate for three subjects [37]. However, no algorithm was presented in this study, no occlusion was considered during the test, and only three subjects were observed and evaluated, which could lead to bias because of personal dietary habits. Disregarding the limitations, this study validated the feasibility of using the distance between hands, head, and plate for intake monitoring. In [52], the angle of the upper lime joint was detected using the skeleton tracking function of MS Kinect and divided into the shoulder, elbow, wrist, and hand. The data were then used for training an SVM to classify the sitting posture, and the number of bites was counted depending on the jaw movement and the distance between the hand and mouth [52].

A similar method was seen in recent research in 2020 for intake counting, implemented on an Intel Core i7 CPU with 8 GB of RAM [51]. This research detected intake by analysing human joint motion during food/drink intake captured by a Kinect depth camera and achieved an average accuracy of 96.2% (also mentioned in Section 4.2). Specifically, the system counted one food intake activity when the hand, wrist and mouth were detected close enough, and the elbow joint and wrist angle exceeded certain thresholds. Moreover, interfering actions, including hand at chin, hand at nose etc., were all considered and analysed [51], which was one of the reasons for the meaningful increase in accuracy.

Except for setting threshold, using classifiers such as Naive Bayes classifier, Haar-Cascade classifier, and SVM mentioned, hidden Markov model (HMM) was another algorithm used. In [41], HMM was used for detecting eating gestures and classifying soup and main dishes in conjunction with an MS Kinect camera. The feature used to indicate a candidate intake activity was the distance between hands and the plate/glass. Unlike the studies that only considered the value of distance, the time duration of the intake movement was also measured and evaluated in this study. This study resulted in 72.7–90% of sensitivity on detection and less than 83% of success rate on classification.

In the dimension of computer vision and machine learning, unsupervised machine learning algorithms, including self-organizing map (SOM) [148], extended SOM [149], and growing neural gas network (GNG) [150] were used for tracking food intake movements [53]. The position of the head and two hands were used to build nodes in the self-organizing neural networks. The best network GNG achieved less than 37 mm mean distance error on the hands and head tracking [53]. Methods built on object detection algorithms were seen in [42], where the colour-tracked skin regions of hands and face over video frames on the combined YCbCr and YIQ colour spaces, and the intaking activities were detected by calculating and evaluating the Euclidean distance between the bounding boxes encircling the tracked skin regions. The results indicated that 90.82% of the correct detecting rate was achieved on around 200 eating episodes. However, neither the occlusion problem nor the clothes colour of subjects was addressed in this study, which could notably influence the result. Recently, eating behaviour, food type, and food amount were detected by a trained model with the video dataset collected by a 360-degree camera [24]. In this pilot experiment, a six-layer CNN (a simplified AlexNet) was trained for recognising hand-to-mouth movements, achieving 70% accuracy, and then extending it to distinguishing the gesture of consuming different foods and using different containers. The proposed method tried to realise food type classification by recognising the gesture of people consuming them, which was different from the previous object detection-based methods. However, only a small amount of data were used in this research, so the training process remained to be conducted on a larger dataset.

Some of the previous research addressed the burden of computing images and videos on food/drink intake activities. While most of the computation happened on a server or offline computer, a micro-control board was used [151] as the computation platform for real-time intake detection, which was based on the joint information of both hand gestures and jaw movement provided by a Kinect.

Compared to third-person methods, the employment of first-person methods for intake action detection was much less. The main technical reason was that using a third-person camera could reduce wearable devices’ high false alarm ratio [51]. However, the feasibility of using a wearable camera for daily activity recording and analysing was tested in [152], which successfully reconstructed daily time usage from wearable cameras. In this study, a mean of 19.2 activities was reported in a day, while 41.1 were revealed by the imaged data captured, proving that first-person cameras can help capture daily activities more accurately than manual reporting. Similarly, a wearable camera was used for recording the activities of users during transportation, with a set of image coding including posture recognition, eating episode detection, food, and beverage type recognition [43]. The specific algorithms and methods were not presented in the paper, but this work evaluated the feasibility of monitoring dietary activities in transportation using a wearable camera.

### 5.4. Intake Amount Estimation

The studies mentioned above were mostly related to intake detection and classification rather than intake amount estimation. However, food volume estimation is another problem to be considered, which is the ‘how much’ problem [73,115,128,153,154,155]. Meal estimation could be realised based on the respective number of intaking gestures for consuming liquid, soup, and meal [41], but the accuracy was not evaluated. Volume estimation based on 3D reconstruction algorithms from images taken by phone was seen in [155], resulting in less than 0.02 inch absolute error for radius estimation (for radius ranging from 0.8 inches to 1.45 inches). Im2Calories was another example, which firstly predicted the distance of each pixel from the camera using a CNN trained on the NYUv2 RGBD dataset, resulting in an average relative error of 0.18 m, which was too high [73]. Then, the depth map was converted to voxel representation for food size estimation, resulting in less than 400 mL absolute volume error [73]. Similarly, a system called FIVR (food intake and voice recognizer) was developed for quantitative nutrition information acquisition from a set of three images and the speech of a user’s meal. Furthermore, 3D reconstruction algorithms were used in this design, reaching a (5.75 ± 3.75)% error in the volume [154]. A CNN was proposed for depth prediction and volume estimation and significantly improved performance with less than 0.2 s runtime, which was 25 times shorter than conventional 3D reconstruction methods [128]. A geometric model for food amount estimation from single-view images was proposed and achieved less than 6% error for energy estimation, but only on the assumption of accurate segmentation and food classification [153]. Stress-log was another system proposed for calorie counts, which achieved 97% accuracy. In this design, 1000 food-related images were collected from Pixabay (an open-access repository) and used to train an object detection application programming in the TensorFlow interface. Then the Firebase Database was used for generating calorie information [115].

As for fluid amount estimation, a design called ‘Playful Bottle’ was proposed, which combined the camera and accelerometer on the phone to realise fluid intake tracking and reminding [156]. The accelerometer was used for drinking action detection, in which case 21.1% of false-positive detections could be caused by shaking the bottle without actually drinking from it. The camera was used to capture images of the liquid amount in the bottle for water level estimation when drinking action was detected, with a 3.86% average error rate over the 16 subjects [156].

## 6. Discussion

Both first-person and third-person methods are faced with viewing occlusion in a free-living environment. However, not many third-person methods were tested in free-living environments compared to first-person methods. For wearable cameras, the camera’s position could change with the body movement and cause incompetent frames; or the camera can be accidentally covered by hair or clothes. For third-person cameras, the occlusion happens when the subjects move to a blind spot or block the expected sight with body parts or clothes, drawing forth to using multiple cameras in the living environment. Therefore, compared to the third-person, the first-person, which can move around with the subject, has the advantage of being individually used in a free-living environment. However, the incontinence of wearing, uncertainty of the wear time and battery sustainability are problems hindering the utilization of first-person cameras. In contrast, as a non-intrusive and almost transparent approach, third-person cameras are more popular for noncompliant groups or people with difficulties using wearable devices, including older adults.

RGB and depth cameras were used as third-person cameras, while only RGB cameras were used as first-person cameras. The reason that no depth camera was ever used as a wearable camera could be the unsuitable size and weight of the device. RGB cameras are commonly used with other non-vision sensors for intake monitoring, potentially improving performance and reducing power consumption. However, in this case, the action detection task was done mainly by non-vision sensors rather than the camera. Depth cameras in third-person methods were primarily used independently, without other non-vision sensors. This could be because it can provide the information that other sensors can provide, including acceleration, distance, angle of pitch, roll, yaw etc. MS Kinect was dominantly used compared to other modules of an RGB-D camera. The reason could be the off-the-shelf coding kit from Microsoft for skeleton extraction and body motion tracking. The fusion of RGB and depth information has been increasingly seen in recent years and has been proven to improve the performance of intake monitoring. Still, it also faces the trade-off between computation, power consumption and accuracy.

The binary classification task was mainly based on first-person images obtained from a wearable RGB camera in the forms of glasses, watch or pendant. The body’s movement could easily cause motion blur and occlusion to the captured image, so obtaining clear intake-related images is the preliminary step for robust and effective intake monitoring in free-living scenarios. Most of the action detection methods used third-person cameras, and MS Kinect was dominantly used, in which RGB information and depth information were used individually or collaboratively. The distance and orientation of different body parts were evaluated to determine actions, such as ‘the distance between mouth to hands’ or ‘the angle between the elbow joint and hand wrist’. The time duration of this movement could also be considered for judgement. However, because bringing hands to the mouth was often seen as a representation of intake, other similar actions, including touching the nose, wiping the mouth, and adjusting glasses, can be easily mistaken as intake action. This has not been thoroughly considered in the existing research. Almost all the vision-based intake amount estimation methods were designed for food/calorie quantification rather than fluid.

In the investigated algorithms, DL methods were the most popular and tended to achieve high performance, while ML methods could be used with DL to boost the accuracy further. However, the training process could be time and energy-consuming. In real-life practice, the simplicity and robustness of the system are essential, and privacy is always an issue. Therefore, if the computation power is in place and the training sample size is sufficient, DL methods are recommended to maximise accuracy, especially if real-time performance is not a requirement. The acceptance of the monitoring technologies could be different depending on the individuals. The methods mentioned in this review will give general solutions to monitoring tasks and can be making instructions for designing personal systems for some special individuals. Limited research was built or evaluated in real-living scenarios.

Regarding privacy preservation, the existing solutions include avoiding taking privacy-sensitive images or manually deleting them during pre-processing. Algorithms for tracking human faces were developed to remove or blur them. Another popular approach was to conduct intake monitoring based only on depth information showing the contour or skeleton of the human without an identifiable face, which complicated the system.

## 7. Conclusions on Research Gaps

This extensive review of Vision-based Methods for Food and Fluid Intake Monitoring: A Literature Review provides the following conclusion and research gaps to drive future direction in this area. A limited paper was found on the drink/non-drink (binary) classification, while there’s a lot for food/non-food classification. This is the preliminary step of identifying intake activities; the interference daily activities (e.g., wiping mouth) were not brought in in current studies to improve the accuracy of the binary classification task. Furthermore, limited papers were found on fluid type classification, much less than food type classification; the drink type included was also limited; the performance of the proposed methods for fluid type classification was lower than what has been achieved on food type classification.

The first-person method was not commonly used for intake action recognition; when an RGB first-person camera was used with non-vision sensors, the action recognition task was mostly conducted by the non-vision sensors rather than the camera. The non-vision sensors used with first-person cameras include an accelerometer, gyroscope, flex sensor, load cell, proximity sensor and IMU. The EMG sensors and microphones were not commonly used but could be an option. Combining first-person RGB cameras with other sensors has the potential to reduce the energy consumption of cameras, extend the use time of batteries, save storage space, and rule out privacy concerns by turning the camera on only when a candidate movement is detected.

In the four tasks mentioned in the paper, third-person methods were mostly used for action recognition rather than other tasks; and the third-person camera was not used collaboratively with non-vision sensors. The result of RGB and depth fusion was promising for intake detection, but the number of papers using this method was limited. The limitations of utilizing first-person cameras for intake monitoring include the occlusion of view, dark and blurry images obtained in poor light conditions, noncompliance in wearing the camera, battery sustainability issues, and privacy issues.

Regarding reducing the concern of privacy and the image data, some studies only used depth information from RGB-D cameras by skeletal tracking. There has been no standalone dataset related to fluid intakes, such as an image dataset for containers or a video dataset for people drinking with different postures, temperatures, containers, and amounts of fluid. Vision-based methods were barely used in fluid intake amount estimation, which is typically done by smart containers, EMG sensors or microphones. The performance of intake monitoring systems proposed in current studies was not adequately tested in a free-living environment.

## Figures and Tables

**Table 1:** Search terms of multiple databases. The denominator in ‘records’ means the number of papers retrieved from the database. The numerator implies the number of documents included in the searching phase for the first round of screening. (The question mark means there is no access to the total number).

Database	Stage	Search Items Used in the Advanced Searching Function	Records
PubMed	Ⅰ	(((fluid[Title/Abstract]) OR (drink[Title/Abstract]) OR (water[Title/Abstract]) OR (liquid[Title/Abstract]) OR (food[Title/Abstract]) OR (nutrition[Title/Abstract]) OR (energy[Title/Abstract]) OR (dietary[Title/Abstract])) AND (((vision[Title/Abstract]) OR (camera[Title/Abstract])) AND ((monitoring[Title/Abstract]) OR (detection[Title/Abstract]) OR (recognition [Title/Abstract])))) AND (intake)	64/64
	Ⅱ	(vision[Title/Abstract] OR camera[Title/Abstract] OR image[Title/Abstract]) AND (action[Title/Abstract] OR gesture[Title/Abstract] OR activity[Title/Abstract] OR motion[Title/Abstract]) AND (detection[Title/Abstract] OR monitoring[Title/Abstract] OR recognition[Title/Abstract]) AND (drink[Text Word] OR water[Text Word] OR liquid[Text Word] OR food[Text Word] OR nutrition[Text Word] OR energy[Text Word] OR dietary[Text Word]) AND (human[Text Word])	126/126
SCOPUS	Ⅰ	TITLE-ABS-KEY (((fluid) OR (drink) OR (water) OR (liquid) OR (food) OR (nutrition) OR (energy) OR (dietary)) AND ((vision) OR (camera)) AND ((monitoring) OR (detection) OR (recognition))) AND (intake) AND (LIMIT-TO (SUBJAREA, “COMP”) OR LIMIT-TO (SUBJAREA, “ENGI”) OR LIMIT-TO (SUBJAREA, “NURS”)) AND (EXCLUDE (SUBJAREA, “MATH”) OR EXCLUDE (SUBJAREA, “AGRI”) OR EXCLUDE (SUBJAREA, “PHYS”) OR EXCLUDE (SUBJAREA, “BIOC”) OR EXCLUDE (SUBJAREA, “MATE”) OR EXCLUDE (SUBJAREA, “CHEM”)) AND (EXCLUDE (SUBJAREA, “PSYC”) OR EXCLUDE (SUBJAREA, “DECI”) OR EXCLUDE (SUBJAREA, “SOCI”) OR EXCLUDE (SUBJAREA, “ENER”) OR EXCLUDE (SUBJAREA, “EART”) OR EXCLUDE (SUBJAREA, “ENVI”))	112/125
	Ⅱ	(TITLE-ABS-KEY ((action OR gesture OR activity OR motion) AND (detection OR monitoring OR recognition) AND (vision OR camera OR image)) AND ALL (human) AND ALL (drink OR water OR liquid OR food OR nutrition OR energy OR dietary)) AND (LIMIT-TO (SUBJAREA, “COMP”) OR LIMIT-TO (SUBJAREA, “ENGI”) OR LIMIT-TO (SUBJAREA, “BIOC”) OR LIMIT-TO (SUBJAREA, “HEAL”) OR LIMIT-TO (SUBJAREA, “NURS”)) AND (EXCLUDE (SUBJAREA, “MATH”) OR EXCLUDE (SUBJAREA, “PHYS”) OR EXCLUDE (SUBJAREA, “MATE”) OR EXCLUDE (SUBJAREA, “CHEM”) OR EXCLUDE (SUBJAREA, “AGRI”) OR EXCLUDE (SUBJAREA, “ENVI”) OR EXCLUDE (SUBJAREA, “SOCI”) OR EXCLUDE (SUBJAREA, “CENG”) OR EXCLUDE (SUBJAREA, “DECI”) OR EXCLUDE (SUBJAREA, “EART”) OR EXCLUDE (SUBJAREA, “ENER”) OR EXCLUDE (SUBJAREA, “PHAR”) OR EXCLUDE (SUBJAREA, “IMMU”) OR EXCLUDE (SUBJAREA, “MULT”) OR EXCLUDE (SUBJAREA, “BUSI”) OR EXCLUDE (SUBJAREA, “PSYC”) OR EXCLUDE (SUBJAREA, “ARTS”) OR EXCLUDE (SUBJAREA, “ECON”) OR EXCLUDE (SUBJAREA, “VETE”) OR EXCLUDE (SUBJAREA, “DENT”)) AND (EXCLUDE (SUBJAREA, “MEDI”) OR EXCLUDE (SUBJAREA, “BIOC”))	100/3334
IEEE Xplore	Ⅰ	(“All Metadata”: fluid OR “All Metadata”: drink OR “All Metadata”: water OR “All Metadata”: liquid OR “All Metadata”: food OR “All Metadata”: nutrition OR “All Metadata”: energy OR “All Metadata”: dietary) AND (“All Metadata”: vision OR “All Metadata”: camera) AND (“All Metadata”: monitoring OR “All Metadata”: detection OR “All Metadata”: recognition) AND (“Full Text and Metadata”: intake)	203/203
	Ⅱ	(“All Metadata”: vision OR “All Metadata”: camera OR “All Metadata”: image) AND (“All Metadata”: action OR “All Metadata”: gesture OR “All Metadata”: activity OR “All Metadata”: motion) AND (“All Metadata”: detection OR “All Metadata”: monitoring OR “All Metadata”: recognition) AND (“Full Text and Metadata”: human) AND (“Full Text and Metadata”: drink OR “Full Text and Metadata”: water OR “Full Text and Metadata”: liquid OR “Full Text and Metadata”: food OR “Full Text and Metadata”: nutrition OR “Full Text and Metadata”: energy OR “Full Text and Metadata”: dietary)	100/11463
ACM Digital Library	Ⅰ	[[[Title: fluid] OR [Title: drink] OR [Title: water] OR [Title: liquid] OR [Title: food] OR [Title: nutrition] OR [Title: energy] OR [Title: dietary]] AND [[Title: vision] OR [Title: camera]] AND [[Title: monitoring] OR [Title: detection] OR [Title: recognition]] AND [[Abstract: or((((fluid] OR [Abstract: drink] OR [Abstract: water] OR [Abstract: liquid] OR [Abstract: food] OR [Abstract: nutrition] OR [Abstract: energy] OR [Abstract: dietary]] AND [[All: vision] OR [All: camera]] AND [[All: monitoring] OR [All: detection] OR [All: recognition]]] OR [All:)))) and (intake)]	100/2983
	Ⅱ	[[Title: action] OR [Title: gesture] OR [Title: activity] OR [Title: motion]] AND [[Title: detection] OR [Title: monitoring] OR [Title: recognition]] AND [[Title: vision] OR [Title: camera] OR [Title: image]] AND [[Abstract: or] OR [[[Abstract: action] OR [Abstract: gesture] OR [Abstract: activity] OR [Abstract: motion]] AND [[Abstract: detection] OR [Abstract: monitoring] OR [Abstract: recognition]] AND [[Abstract: vision] OR [Abstract: camera] OR [Abstract: image]]]] AND [[Full Text: drink] OR [Full Text: water] OR [Full Text: liquid] OR [Full Text: food] OR [Full Text: nutrition] OR [Full Text: energy] OR [Full Text: dietary]] AND [Full Text: human]	38/38
Web of Science	Ⅰ	In Topic: fluid OR drink OR water OR liquid OR food OR nutrition OR energy OR dietary And in Topic: vision OR camera And in Topic: monitoring OR detection OR recognition And in All Fields: intake	214/214
	Ⅱ	((((TS = (action OR gesture OR activity OR motion)) AND TS= (detection OR monitoring OR recognition)) AND TS = (vision OR camera OR image)) AND ALL = (drink OR water OR liquid OR food OR nutrition OR energy OR dietary)) AND ALL = (human)	100/1295
Google Scholar	Ⅰ	((fluid) OR (drink) OR (water) OR (liquid) OR (food) OR (nutrition) OR (energy) OR (dietary)) AND ((vision) OR (camera)) AND ((monitoring) OR (detection) OR (recognition)) AND (intake)	50/?
	Ⅱ	(Action OR gesture OR activity OR motion) AND (detection OR monitoring OR recognition) AND (vision OR camera OR image) AND (human) AND (drink OR water OR liquid OR food OR nutrition OR energy OR dietary)	50/?
Total number of retrieved records			1257
Number after duplicated items removed			1001

**Table 2:** Summary of the studies using both RGB and depth information for intake monitoring.

References	Tasks				Usage of the Devices					Devices Model	Methods (Algorithms)	Assessment
	Binary Classification	Type Classification	Intake Action Recognition	Amount Estimation	Single Camera	Multiple Camera	Collaborative with Non-Vision	Fusion	Placement of the Camera			
Cippitelli et al., 2016 [62]			**✓**		**✓**			**✓**	mounted on ceiling, top-down view	Kinect	1. SOM(Self-Organized Map) on the defined skeleton model for movement tracking; 2. Glass detection (circular property) from RGB stream based on Hough function; 3. RGB-D fusion based on stereo calibration theory.	Correct rate of 98.3% for intake action classification.
Iizuka et al., 2018 [68]		**✓**		**✓**	**✓**				top-down view	Intel RealSense F200	1. Seperalty using a 3-layer perceptron NN and a VGG16 network-based CNN with data augmentation for meal type classification; 2. Felzenszwalb’s segmentation algorithm for segmenting each food element; 3. Using the weight density information for each meal type to estimate the weight.	1. More than 90% recognition accuracy on meal type; 2. About 4% error rate for estimating the weight of each meal elements.
Zhang et al., 2018 [69]	Orientation estimation					**✓**			one from upper above and one from front view pointing at the subject	Kinect 2.0	1. CNN for container recognition; 2. Region growing algorithm for object segmentation from point cloud on color and depth information; 3. PCA (Principal Component Analysis) for object orientation estimation.	Average of 90.3% success rate when detecting and estimating the orientation of handleless cups.
Lo et al., 2019 [65]				**✓**	**✓**		**✓**		from any convenient viewing angle	RealSense	1. Unsupervised segmentation method for segmenting each food item; 2. Point completion deep Neural Network for 3D reconstruction and portion size estimation of food items.	Up to 95.41% accuracy for food volume estimation.
Chang et al., 2019 [70]			**✓**		**✓**			**✓**	from various viewpoints	Kinect 2.0	1. Human region detection based on SVM from histogram of oriented gradient features extracted from the RGB streams; 2. Farnebäck method for obtaining corresponding frames of the optical flow video from RGB stream; 3. Three modified 3D CNNs to extract the spatiotemporal features of human actions and recognize them.	Average human action recognition rate of 96.4%.
Gambi et al., 2020 [71]			**✓**		**✓**				mounted on ceiling, top-down view	Kinect v1	1. An improved SOM Ex algorithm to trace the movements of the subject using a model of 50 nodes; 2. Setting threshold for distance of the joints to detect the start frame (SF) and the end frame (EF) for eating.	Action detection MAE(mean absolute error) of 0.424% and a MRE(mean relative error) of 5.044%.
Raju et al., 2022 [72]				**✓**		**✓**			all pointing at the food	OV5640 cameras, Panasonic PIR sensor, IR dot projector	1. Semiglobal matching (SGM) for stereo matching to find correspondence between the pixels of the stereo images; 2.3D reconstruction for a dense disparity map and then point cloud to calculate the volume.	Average accuracy of 94.4% on food portion sizes estimation.
